# Nuclear Factor Kappa B Activation Occurs in the Amnion Prior to Labour Onset and Modulates the Expression of Numerous Labour Associated Genes

**DOI:** 10.1371/journal.pone.0034707

**Published:** 2012-04-02

**Authors:** Sheri Lim, David A. MacIntyre, Yun S. Lee, Shirin Khanjani, Vasso Terzidou, T. G. Teoh, Phillip R. Bennett

**Affiliations:** 1 Imperial College Parturition Research Group, Institute of Reproduction and Developmental Biology, Imperial College London, London, United Kingdom; 2 Department of Obstetrics and Gynaecology, St. Mary's Hospital, London, United Kingdom; VU University Medical Center, Netherlands

## Abstract

**Background:**

Prior to the onset of human labour there is an increase in the synthesis of prostaglandins, cytokines and chemokines in the fetal membranes, particular the amnion. This is associated with activation of the transcription factor nuclear factor kappa B (NFκB). In this study we characterised the level of NFκB activity in amnion epithelial cells as a measure of amnion activation in samples collected from women undergoing caesarean section at 39 weeks gestation prior to the onset of labour.

**Methodology/Principal Findings:**

We found that a proportion of women exhibit low or moderate NFκB activity while other women exhibit high levels of NFκB activity (n = 12). This activation process does not appear to involve classical pathways of NFκB activation but rather is correlated with an increase in nuclear p65-Rel-B dimers. To identify the full range of genes upregulated in association with amnion activation, microarray analysis was performed on carefully characterised non-activated amnion (n = 3) samples and compared to activated samples (n = 3). A total of 919 genes were upregulated in response to amnion activation including numerous inflammatory genes such cyclooxygenase-2 (COX-2, 44-fold), interleukin 8 (IL-8, 6-fold), IL-1 receptor accessory protein (IL-1RAP, 4.5-fold), thrombospondin 1 (TSP-1, 3-fold) and, unexpectedly, oxytocin receptor (OTR, 24-fold). Ingenuity Pathway Analysis of the microarray data reveal the two main gene networks activated concurrently with amnion activation are i) cell death, cancer and morphology and ii) cell cycle, embryonic development and tissue development.

**Conclusions/Significance:**

Our results indicate that assessment of amnion NFκB activation is critical for accurate sample classification and subsequent interpretation of data. Collectively, our data suggest amnion activation is largely an inflammatory event that occurs in the amnion epithelial layer as a prelude to the onset of labour.

## Introduction

Amnion epithelial cells contain large stores of the prostaglandin precursor arachidonic acid (AA) [1], [2] and synthesis of its metabolites, especially prostaglandin (PG) E2, increases dramatically at the onset of labour [3]. This increase is associated with a reduction in prostaglandin dehydrogenase activity in the chorion [4] that facilitates PG-modulated cervical ripening and uterine contractility. At labour, PG synthesis in amnion is principally via the inducible cyclo-oxygenase enzyme (COX-2) [5], [6], [7], [8].

The amnion is an important source of pro-inflammatory chemokines and cytokines (eg. IL-8 and IL-1β), the levels of which increase in amnion epithelial cells with the onset of labour [9]. IL-8 acts by attracting neutrophils into the uterine cervix and myometrium [10], [11] that subsequently contribute to fetal membrane remodelling and cervical ripening by release of metalloproteinases, such as MMP-8 (neutrophil elastase) [12]. IL-1β elicits a ‘positive feed-forward’ response to further increase IL-8 synthesis and PG synthesis through upregulation of COX-2. Both COX-2 and IL-8 are regulated in amnion by the transcription factor nuclear factor kappa B (NFκB) [13], [14] and IL-1β is NFκB-regulated in a range of cell types [15], [16], [17].

NF-κB is an inducible transcription factor consisting of DNA binding dimers of various subunit combinations that determine functionality. The NF-κB subunits are derived from transcripts of five genes: NF-κB1 (encoding p50 and its precursor p105), NF-κB2 (encoding p52 and its precursor p100), RelA (p65), c-Rel, and Rel-B. Rel-B, p65, and c-Rel contain C-terminal non-homologous transactivation domains (TADs) that facilitate kinase-induced transcriptional activation. Proteolytic processing of p105 and p100 via a ubiquitin-proteasome pathway [18] leads to the formation of p50 and p52 subunits that lack TADs and are thus considered inhibitory [19]. In cells with basal or no NFκB activity, NF-κB dimers are retained in the cytoplasm in an inactive form by binding to IκB proteins, IκBα, IκBβ, and IκBε. NF-κB can also be sequestered in the cytoplasm by the p105 and p100 precursor proteins where they act as IκB proteins. Classically, NFκB activation is provoked through the activation of cell surface receptors such as IL-1, TNF or Toll-like receptors, inducing a signalling cascade which converges on and activates the IκB kinase (IKK) complex, consisting of the regulatory scaffold protein NF-κB essential modulator (NEMO) and IKKα and IKKβ kinases [20]. Activation of IKK leads to phosphorylation of the NFκB inhibitor IKBα and its subsequent ubiquitination and degradation [21]. The degradation of ubiquitinated IκB releases NF-κB dimers from the cytoplasmic IκB/NF-κB complex, allowing nuclear translocation of NF-κB to specific recognition elements in target gene promoters to drive gene expression [22], [23], [24].

In earlier studies conducted at a time (1999–2000) when elective caesarean sections were routinely performed two weeks prior to the estimated date of delivery, we found that amnion epithelial cells obtained following spontaneous labour and vaginal delivery exhibited consistent, high levels of nuclear NFκB-DNA binding and transcriptional activity which could not be further stimulated by incubation with IL-1β [13]. In contrast, amnion cells from cultured placentas collected following elective caesarean section before the onset of labour typically displayed low level nuclear NFκB-DNA binding and transcriptional activity that could be stimulated with IL-1β to levels similar to that seen in post-labour samples [13], [17]. This pattern parallels data concerning arachidonic acid metabolism in pre-labour amnion cells in which PG synthesis is low but can be stimulated, whilst in post-labour cells PG synthesis is high and cannot be stimulated significantly further [25].

Following changes to national guidelines, elective caesarean section in the UK is now routinely performed after 39 weeks, closer to the likely time of the onset of labour. We now find that a subset of amnion samples taken from women following pre-labour elective caesarean section show levels of activation similar to those seen following the onset of labour (presumably because they are biochemically closer to the onset of clinical labour). This provides the opportunity to determine the effects of amnion activation at term, independently of any effects that labour and delivery might have upon the amnion. In this study levels of NFκB activity was used as a measurement of amnion activation. To examine which genes are regulated in association with amnion activation, samples were carefully categorised into two groups- i) low activation or ii) high activation, and gene differences between the two determined using microarray analysis.

## Results

**Figure 1:**
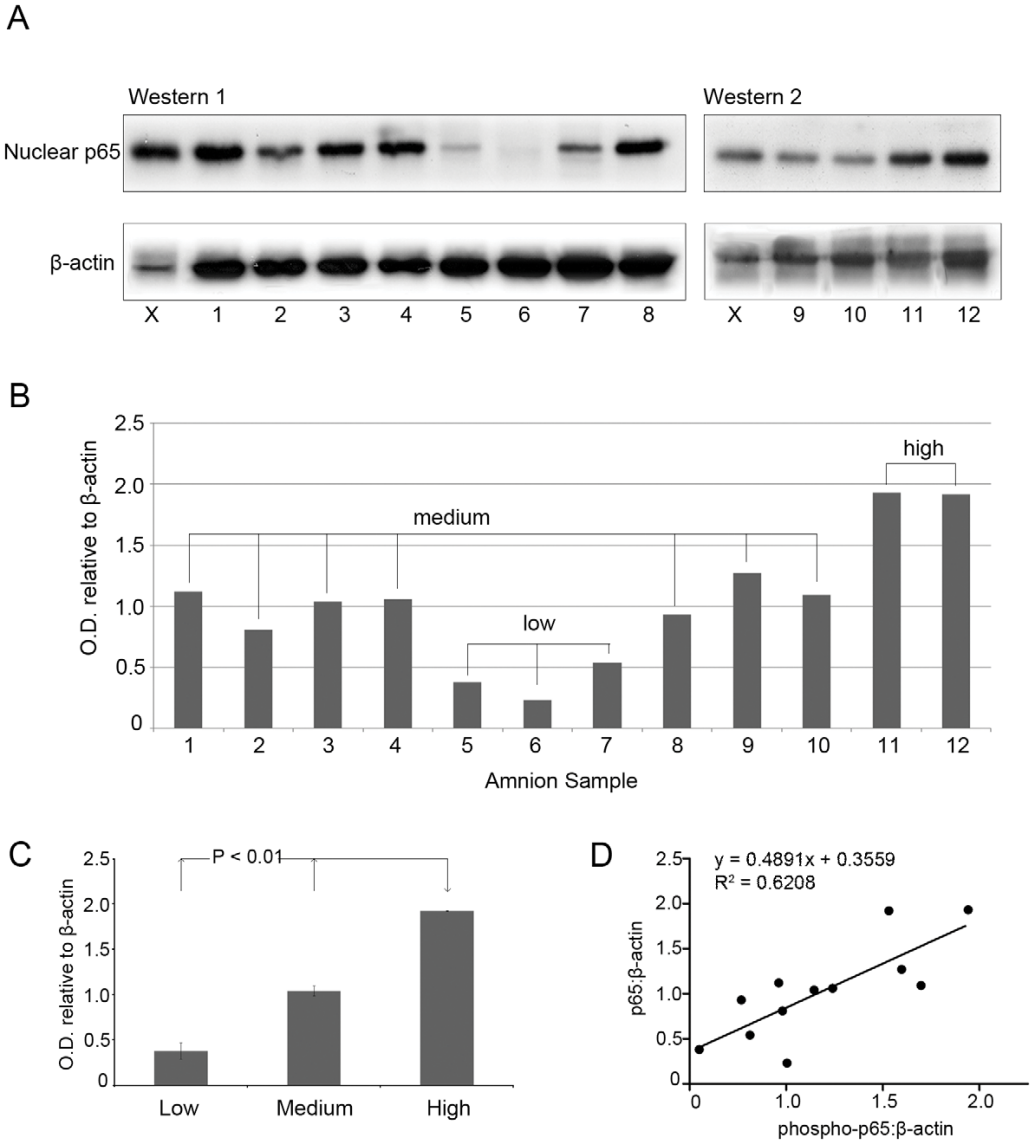
Characterisation of amnion NFκB activity by western blotting. Primary, pre-labour amnion epithelial cells were derived from 12 women undergoing caesarean section. Protein levels of activated NFκB were examined in each sample by immunoblotting for p65 in nuclear extracts, which were then normalised to β-actin (**A**). Following densitometric analysis, samples were categorised into three groups based upon their level of NFκB activation – low, medium and high (**B**). The low NFκB activation group consisted of three samples, the medium of seven samples and the high activation group consisted of two samples. Gel to gel variation was accounted for by loading equal volume of a control sample (X) on each gel. ANOVA revealed significant differences between protein levels of nuclear p65 in the three classified groups (**C**). Levels of nuclear p65 were also shown to correlate with levels of nuclear phosphorylated p65 in a linear fashion (R^2^ = 0.6205).

NFκB plays a critical role in fetal membrane activation and the regulation of labour associated proteins such as COX-2 and IL-8. We hypothesised that the activation of NFκB should therefore occur just prior to the onset of labour. To test this, western blot analysis of primary cell cultures derived from 12 individual, term, pre-labour samples was performed to examine nuclear protein levels of p65 in these samples. Nuclear p65 levels varied markedly between samples (Figure 1A). Three distinct groups could be distinguished which were subsequently divided upon their relative expression levels of nuclear p65 as follows; i) low nuclear p65 concentrations representing low-level NFκB activation and non-activated amnion (<0.6 a.u. nuclear p65: β-actin), ii) medium NFκB activation (>0.6 a.u. and <1.4 a.u. nuclear p65: β-actin) and iii) high NFκB activation (>1.4 a.u. nuclear p65:β-actin; Figures 1B and 1C). Similarly, immunoblotting for nuclear phosphorylated p65 showed variable levels in the 12 samples examined. Levels of nuclear p65 were highly correlated to levels of nuclear phosphorylated p65 indicative of the phosphorylation-dependant translocation of p65 to the nucleus (Figure 1D).

**Figure 2:**
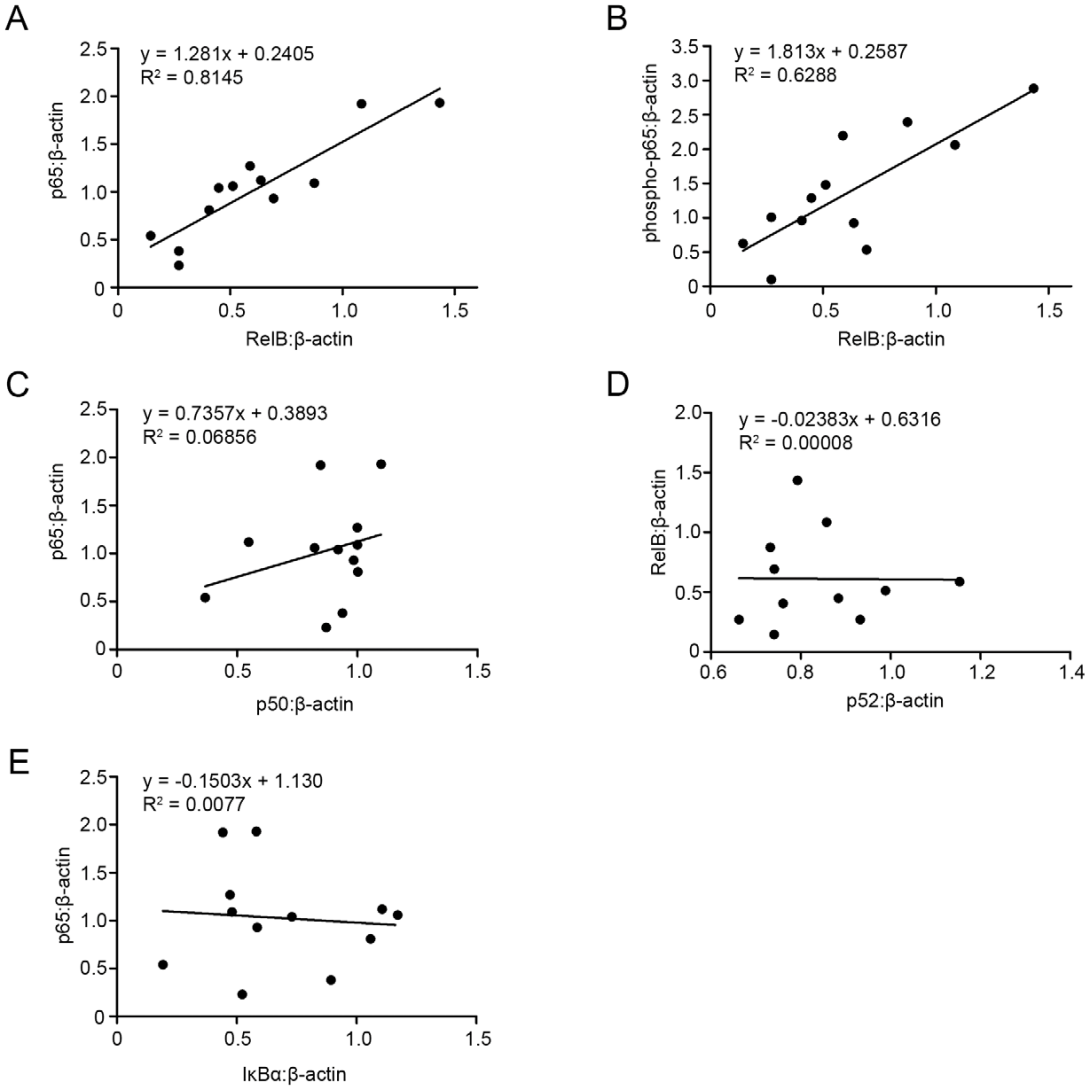
Correlations between key components of the canonical, non-canonical and atypical NFκB signalling pathways. Protein levels of activated NFκB were examined in each sample by immunoblotting as previously described. Levels of both nuclear p65 and nuclear phospho-p65 were shown to correlate highly with nuclear levels with Rel-B (A and B, R^2^ = 0.8145 and R^2^ = 0.6288 respectively). No correlation was detected between nuclear levels of p65 and p50 (C, R^2^ = 0.06856), nuclear Rel-B and p52 (D, R^2^ = 0.00008) or nuclear p65 and IκBα (E, R^2^ = 0.0077). Collectively these results suggest that neither the canonical, non-canonical nor the atypical signalling pathways are responsible for the observed differences in NFκB activation.

The high correlation observed between nuclear p65 and pp65 was suggestive of canonical activation of NFκB, yet high levels of nuclear Rel-B were also detected in the samples (Figure 2A). High concentrations of nuclear Rel-B were strongly correlated with high levels of both nuclear p65 and phosphorylated p65, whereas there was a poor correlation between nuclear p65 and nuclear p50, indicative of non-canonical activation of NFκB. (Figures 2B and 2C). Levels of p52, typically dimerized to Rel-B during non-canonical NFκB activation, were not correlated with nuclear p65 or nuclear Rel-B levels (Fig. 2D). No positive correlation was observed between cytoplasmic IκBα with either nuclear p65 or phosphorylated p65 (Fig. 2E).

**Figure 3:**
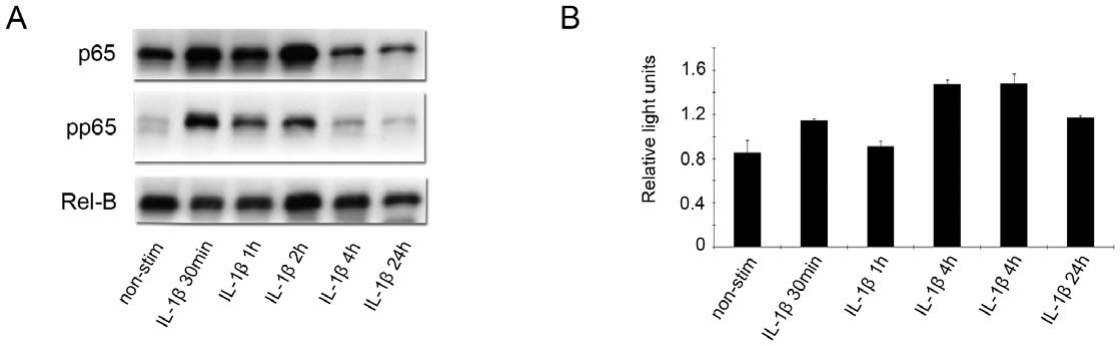
Interactions between p65 and Rel-B exist in pre-labour, human amnion. (**A**) Whole cell lysate from unstimulated and IL-1β stimulated pre-labour amnion epithelial cells was incubated p65 conjugated beads. The lysate was recaptured under denaturing conditions and Western immunoblotting performed with either anti-p65, anti-phospho-p65 or anti-Rel-B antibodies. Non-stimulated amnion was shown to contain both p65-pp65 and to a greater extent, p65-Rel-B dimers. When stimulated with IL-1β, p65-pp65 dimer levels increased maximally at 30 min and then decreased gradually over 24 h. Dimers of p65-Rel-B were maintained at high levels throughout the time series. (**B**) Non-radioactive DNA binding assay kit (TRANSAM perbioscience) measuring the binding of Rel-B to the NFκB consensus binding sequence in response to IL-1β showed an increase from the unstimulated state at 30 min before dropping slightly 1 h. Binding peaked at 4 h before again subsiding at 24 h.

Since a strong correlation of nuclear Rel-B with nuclear p65 and phosporylated p65 was consistently observed, the possibility that p65 and Rel-B subunits may themselves physically interact was investigated. Immunoprecipitation studies using anti-p65 antibody were performed in both non-stimulated and IL-1β-stimulated pre-labour, primary cultured amnion epithelial cells (Figure 3A). Immunoblotting using anti-pp65 revealed the presence of complexes containing pp65 in non-stimulated amnion cells. Upon stimulation, concentrations of complexes containing pp65 increased maximally at 30 min and then gradually reduced to 24 h. Dimers of p65-Rel-B were consistently high in stimulated cells through the time course of the experiment. Binding of Rel-B to the NFκB consensus sequence as assessed using a non-radioactive DNA binding assay was increased after 30 min before dropping slightly after 1 h. Peak binding was reached at 4 h before dipping again at 6 h (Figure 3B).

**Figure 4:**
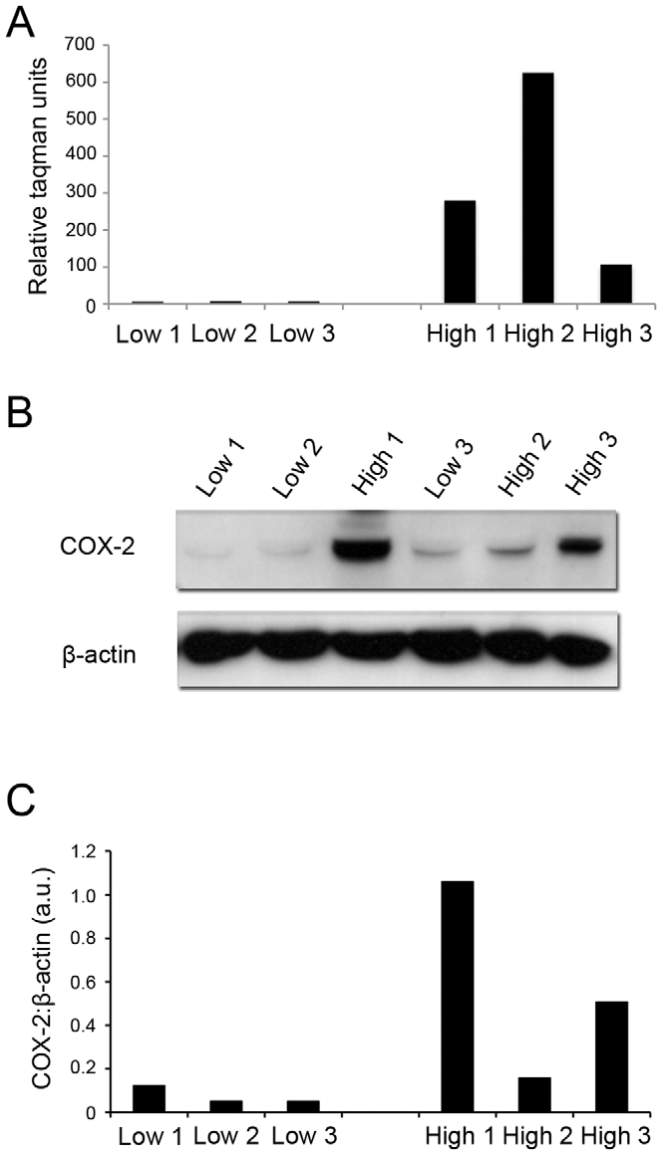
Characterisation of amnion activation for microarray analysis. Prior to microarray analysis, levels of COX-2 mRNA (**A**) and COX-2 protein were assessed in 6 amnion samples by western blotting (**B**) and subsequent densitometric assessment of immuno-reactive bands (**C**). Three samples characteristically displayed high levels of both COX-2 gene and protein consistent with highly activated amnion. Three samples displaying low levels of COX-2 were also selected as non-activated samples. The six characterised samples were subsequently used for microarray analysis to examine genes involved in amnion activation.

**Figure 5:**
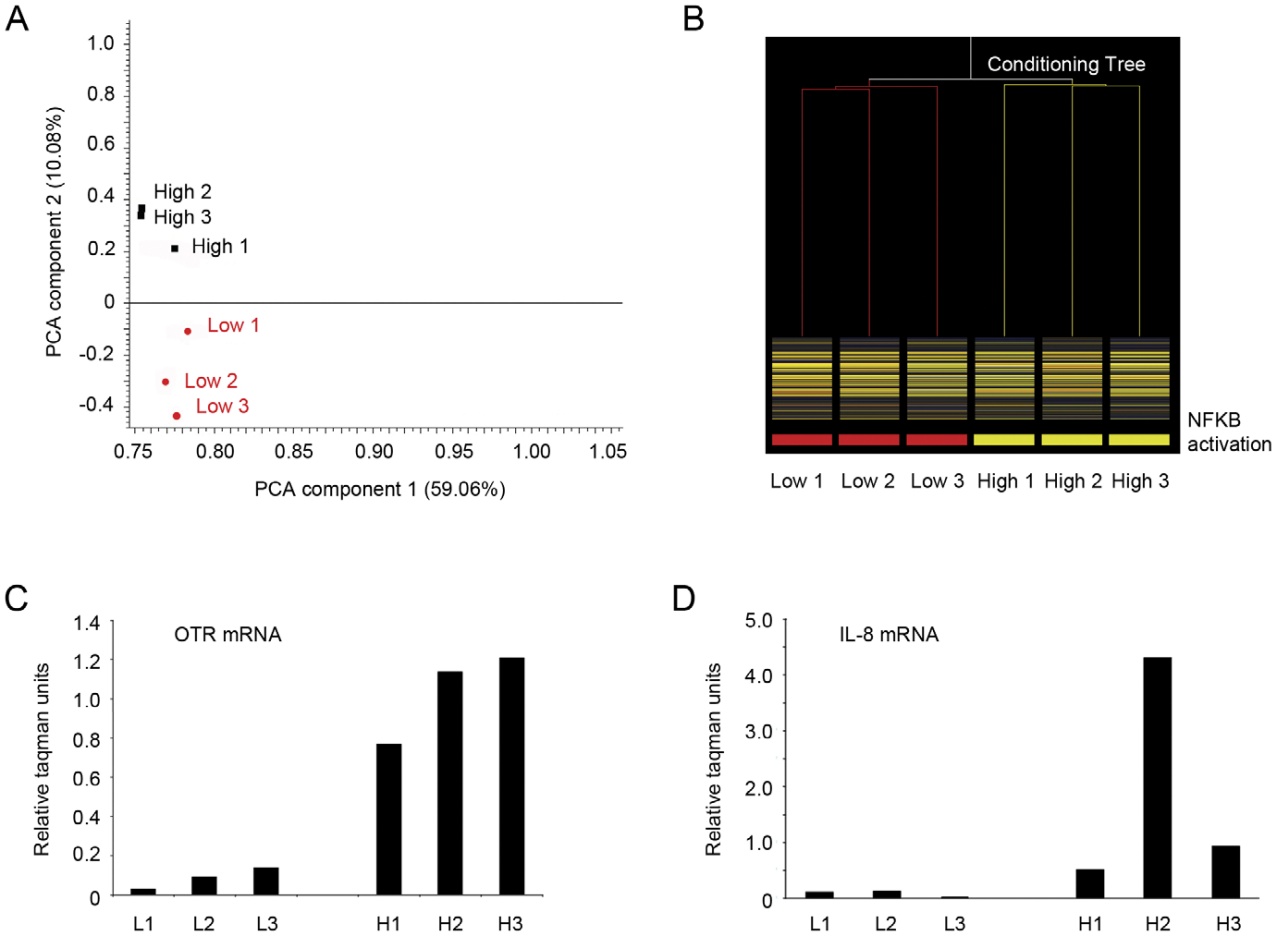
Validation of microarray data. (**A**) Gene data derived from the microarray analysis was firstly examined using unsupervised principal components analysis (PCA). Inspection of the PCA score plot revealed clear first component separation of activated (yellow) and low-activated (red) amnion samples. (**B**) Similarly, hierarchical clustering demonstrated grouping within both the activated and non-activated amnion groups. Collectively these results indicate that gene expression profiles of activated samples are similar to each other, yet distinct to non-activated samples. OTR (**C**) and IL-8 (**D**) mRNA levels were then assessed in 6 amnion samples post microarray analysis. Consistent with the microarray data, three samples displayed low levels of OTR and IL-8 mRNA whilst the other three samples displayed characteristically high levels indicative of amnion activation.

Prior to microarray analysis, three samples containing high levels of NFkB activation were further characterised by measuring COX-2 expression and COX-2 protein levels. Three samples with low levels of NFkB activation and low COX-2 expression were classified as non-activated along with 3 additional highly-activated samples for microarray analysis (Figure 4). Whole genome analysis using Affymetrix U133 arrays was then performed on non-activated (n = 3) and activated amnion samples (n = 3). Unsupervised multivariate statistical analyses approaches in the form of PCA and hierarchical clustering were performed on the gene data to examine underlying variance and correlation in the gene expression data (Figure 5A and 5B). Both approaches revealed clear clustering of activated and non-activated amnion samples indicating that the pre-array molecular characterisation of the samples was representative of the underlying gene expression differences between the sample groups.

Of the 19198 genes examined using the microarray analysis, a total of 919 genes where found to be significantly (*P*<0.05) increased in activated amnion samples compared to non-activated samples. A list of the top 20 most up-regulated genes present in activated amnion was determined (Table 1) with the highest fold changes detected in COX-2 (×44.4), OTR (×24.1), chromosome 10 open reading frame (×19.6), integrin A2 (×17.7) and dimethlyarginine dimethlyaminohydrolase 1 (×16). A directed search for inflammatory response genes yielded COX-2, IL-8 (×6.2), IL1-RAP (×4.5), thrombospondin (×2.7), monoglyceride lipase (2.0), nuclear transcription factor X box binding (1.9), CD40 TNF receptor superfamily member 5 (×1.7) and macrophage migration inhibition factor (×1.7; Table 2). As a further validation of the microarray data, mRNA levels of OTR and IL-8 measured using real-time PCR were found to be significantly increased in the highly activated amnion samples (Figure 5C and 5D).

**Table 1:** List of top 20 genes significantly upregulated in activated amnion.

Rank	Fold Change	Gene Name	Description	Genbank ID	Gene Ontology Biological Process
1	44.4	PTGS2	prostaglandin-endoperoxide synthase 2 (cyclooxygenase 2)	NM_000963	prostaglandin biosynthetic process, fatty acid biosynthetic process, prostaglandin metabolic process, inflammatory response
2	24.1	OXTR	oxytocin receptor	NM_000916	muscle contraction, signal transduction, cell surface receptor linked signal transduction, G-protein coupled receptor protein signaling pathway
3	19.6	C10orf47	chromosome 10 open reading frame 47	AI640157	—
4	17.7	ITGA2	integrin, alpha 2	N95414	cell adhesion, cell-matrix adhesion, integrin-mediated signaling pathway
5	16.0	DDAH1	dimethylarginine dimethylaminohydrolase 1	AL078459	arginine catabolic process, nitric oxide mediated signal transduction
6	14.9	CTGF	connective tissue growth factor	M92934	cartilage condensation, ossification, angiogenesis
7	14.7	PLAUR	plasminogen activator, urokinase receptor	U08839	cell motility, chemotaxis, signal transduction, cell surface receptor linked signal transduction
8	12.5	MAG1	lung cancer metastasis-associated protein	BC006236	metabolic process
9	12.3	—	—	M19154	—
10	11.7	SPRR1B	small proline-rich protein 1B	NM_003125	epidermis development, peptide cross-linking, keratinocyte differentiation
11	10.9	DUSP4	dual specificity phosphatase 4	NM_001394	regulation of progression through cell cycle, MAPKKK cascade, protein amino acid dephosphorylation
12	10.5	—	Homo sapiens, clone IMAGE:3881549, mRNA	BE222344	—
13	10.0	CYR61	cysteine-rich, angiogenic inducer, 61	AF003114	regulation of cell growth, patterning of blood vessels, chemotaxis, cell adhesion, cell proliferation
14	9.8	MAFF	v-maf	AL021977	in utero embryonic development, transcription, regulation of transcription, DNA-dependent
15	9.5	THBD	thrombomodulin	AW119113	pregnancy, blood coagulation, embryonic development
16	9.4	CA2	carbonic anhydrase II	M36532	morphogenesis of an epithelium, one-carbon compound metabolic process, carbon dioxide transport
17	9.2	HEG1	HEG homolog 1 (zebrafish)	AI148659	—
18	9.0	HEG1	HEG homolog 1 (zebrafish)	AA121502	—
19	8.7	KRT6B	keratin 6B	L42612	cytoskeleton organization and biogenesis, ectoderm development
20	8.7	LDLR	low density lipoprotein receptor	NM_000527	protein amino acid O-linked glycosylation, lipid metabolic process

In total, 919 genes were significantly (*P*<0.05) upregulated in activated amnion samples. The top 20 genes with highest fold change are listed (*P*<0.01 for all genes).

**Table 2:** Inflammatory genes significantly upregulated in activated amnion.

Rank	Fold Change	Gene Name	Description	Genbank	GO Biological Process
1	44.4	PTGS2	prostaglandin-endoperoxide synthase 2 (cyclooxygenase 2)	NM_000963	prostaglandin biosynthetic process, fatty acid biosynthetic process, prostaglandin metabolic process, inflammatory response
2	6.3	IL8	interleukin 8	NM_000584	angiogenesis, cell motility, inflammatory response, immune response, cell adhesion signal transduction, G-protein coupled receptor protein signaling pathway
3	4.5	IL1RAP	interleukin 1 receptor accessory protein	AF167343	protein complex assembly, inflammatory response, immune response
4	2.7	THBS1	thrombospondin 1	AW956580	cell motility, inflammatory response, cell adhesion, multicellular organismal development, negative regulation of angiogenesis
5	2	MGC	monoglyceride lipase	BC006230	lipid metabolic process, aromatic compound metabolic process, inflammatory response
6	1.9	NFX1	nuclear transcription factor, X-box binding 1	NM_002504	negative regulation of transcription from RNA polymerase II promoter, transcription, regulation of transcription DNA dependent, inflammatory response, ubiquitin cycle
7	1.7	-	CD40 molecule, TNF receptor superfamily member 5	BF654114	protein complex assembly, inflammatory response, NF-kappaB cascade, immune response
8	1.7	MMIF	macrophage migration inhibitory factor	NM_002415	prostaglandin biosynthetic process, inflammatory response, cell surface receptor linked signal transduction, cell proliferation

A list for inflammatory genes upregulated in activated amnion samples compared to non-activated samples was generated. In total, 8 significant inflammatory genes were identified (*P*<0.01).

**Figure 6:**
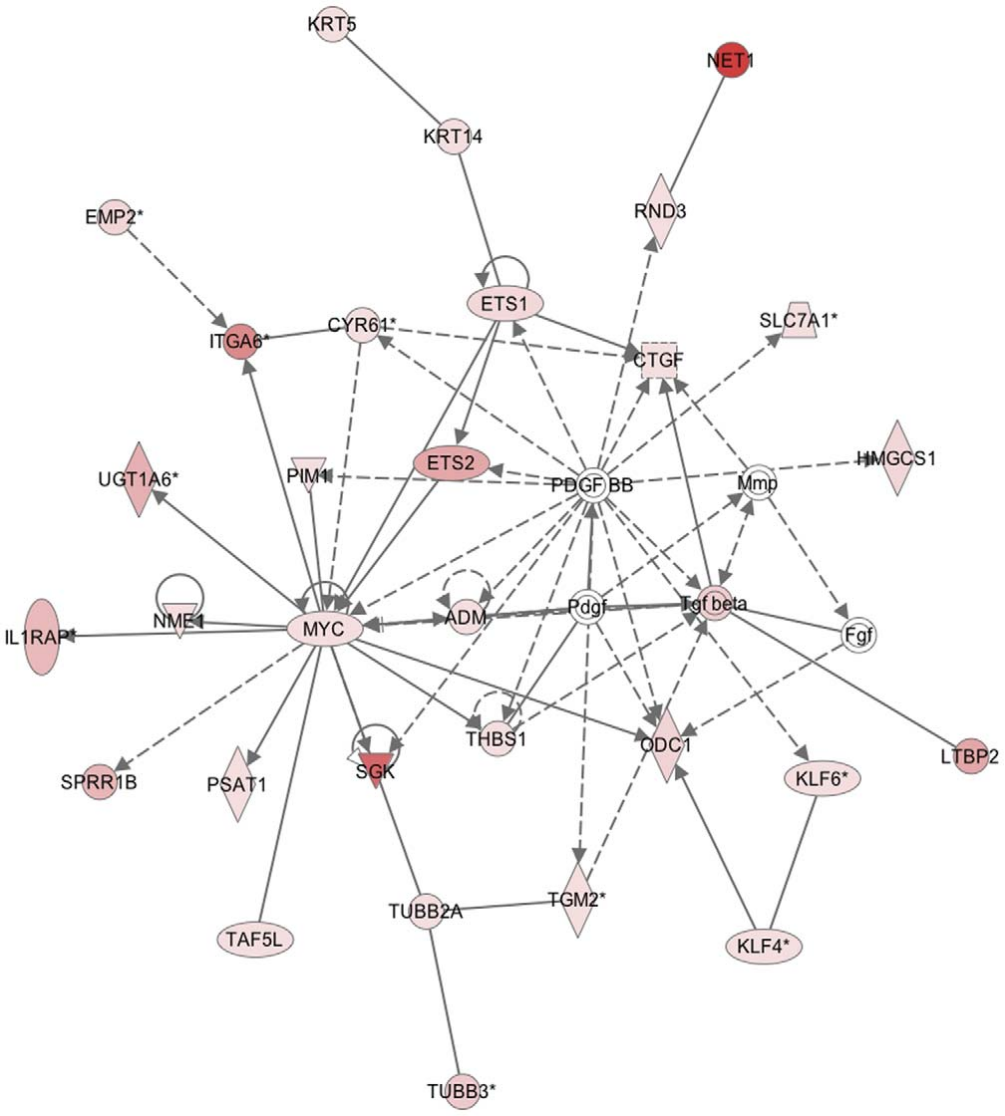
Ingenuity Pathway Analysis (IPA) of gene pathways driving amnion activation- network map 1. Network one comprising of upregulated genes (>2.5-fold) involved predominately in cell death, cancer and morphology. A total of 25 focus genes were used in the construction of the network. ADM (adrenomedullin/progesterone biosynthetic process), CTGF (connective tissue growth factor/angiogenesis), CYR61 (cysteine-rich angiogenic inducer/patterning of blood vessels), EMP2 (epithelial membrane protein 2/cell death), ETS1 (v-ets erythroblastosis virus E26 oncogene homolog 1 (avian)/DNA dependent transcription), ETS2 (DNA dependent transcription), HMGCS1 (transporter 2, ATP-binding cassette, sub-family B (MDR/TAP)/protein complex assembly), IL1RAP (interleukin 1 receptor accessory protein/transmembrane receptor activity involved in the inflammatory response), ITGA6 (integrin, alpha 6/cell adhesion and calcium ion binding), KLF4 (Kruppel-like factor 4 (gut)/DNA dependent transcription and nucleic acid binding), KLF6 (Kruppel-like factor 6/DNA dependent transcription and nucleic acid binding), NET1 (neuroepithelial cell transforming gene 1/regulation of Rho protein signal transduction), NME1 (non-metastatic cells 1, protein (NM23A)/nucleic acid binding), ODC1 (ornithine decarboxylase 1/polyamine biosynthetic process), PIM1 (pim-1 oncogene/protein serine/threonine kinase activity), PSAT1 (phosphoserine aminotransferase 1/phosphoserine transaminase activity), RND3 (Rho family GTPase 3/cell adhesion), SGK (serum/glucocorticoid regulated kinase/serine/threonine kinase activity), SLC7A1 (solute carrier family 7, (cationic amino acid transporter, y+ system) member 11/amino acid transport activity), SPRR1B (small proline-rich protein 1B (cornifin)/epidermis development), TAF5L (TAF5-like RNA polymerase II, p300/CBP-associated factor (PCAF)-associated factor, 65 kDa/DNA dependent transcription), THBS1 (Thrombospondin 1/cell motility, adhesion in response to inflammation), TUBB3 (tubulin, beta 3/microtubule-based movement), TUBB2A (tubulin, beta 2A/microtubule-based movement), UGT1A6 (UDP glucuronosyltransferase 1 family/xenobiotic metabolic process).

**Figure 7:**
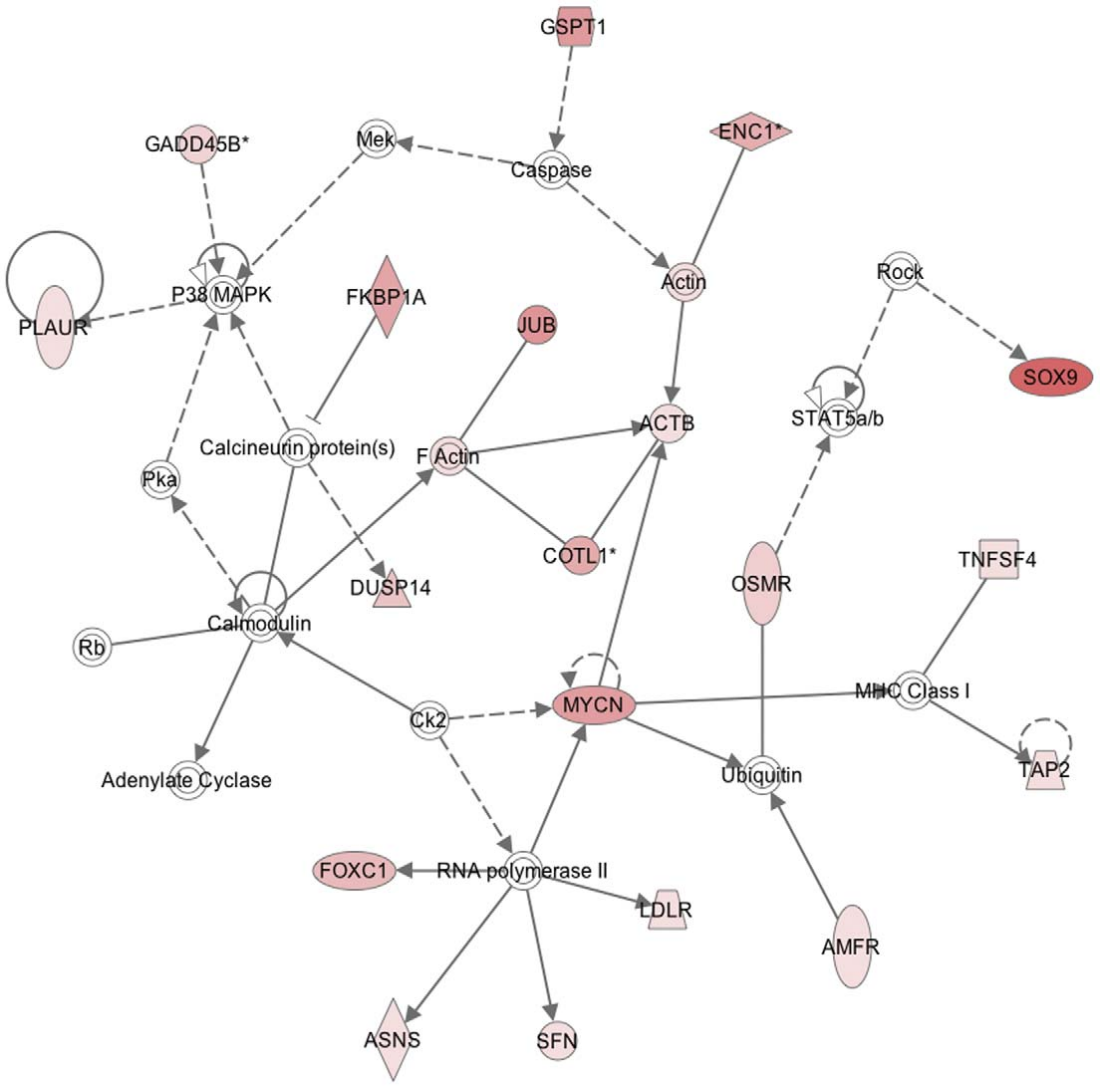
IPA of gene pathways driving amnion activation- network map 2. Network two incorporated 17 focus genes (upregulated >2.5-fold) involved in cell cycle, embryonic development and tissue development. ACTB (actin, beta, cell motility), AMFR (autocrine motility factor receptor, ubiquitin cycle), ASNS (asparagine synthetase, asparagines biosynthetic pathway), Caspase (caspase recruitment domain family, member 10, activation of NFκB inducing kinase), COTL1 (coactosin-like 1 (Dictyostelium), carbohydrate metabolic process), DUSP14 (dual specificity phosphatase 14, protein amino acid dephosphorylation), ENC1 (ectodermal-neural cortex (with BTB-like domain, actin binding), FOXC1 (forkhead box C1, transcription factor activity), GADD45B (growth arrest and DNA-damage-inducible, beta, activation of MAPKK activity), GSPT1 (G1 to S phase transition 1, transition of mitotic cell cycle, translation), JUB (jub, ajuba homolog (Xenopus laevis), zinc ion binding), LDLR (low density lipoprotein receptor (familial hypercholesterolemia), lipid metabolic process), MYCN (v-myc myelocytomatosis viral related oncogene, neuroblastoma derived (avian), regulation of transcription), PLAUR (plasminogen activator, urokinase receptor, cell motility), SFN (stratifin, negative regulation of protein kinase activity), SOX9 (SRY (sex determining region Y)-box 9 (campomelic dysplasia, autosomal sex-reversal), cell fate specification), TNFSF4 (tumor necrosis factor (ligand) superfamily, member 4 (tax-transcriptionally activated glycoprotein 1, 34 kDa), immune response, cytokine activity).

To identify pathways in which the identified gene expression changes may be implicated, canonical pathway and gene ontology analysis were performed using IPA. The two main networks identified were i) cell death, cancer and morphology (25 focus genes- Figure 6) and ii) cell cycle, embryonic development and tissue development (17 focus genes- Figure 7).

## Discussion

We have previously reported that the degree of amnion activation, as defined by levels of nuclear NFκB-p65, in pre-labour amnion cells is highly variable [13]. Following changes to national guidelines, elective caesarean section in the UK is now routinely performed after 39 weeks, closer to the likely time of the onset of labour. In this study we examined amnion activation in cells derived from pre-labour samples collected close to term. A subset of these women exhibited low or moderate levels of NFκB activity whilst others had high activity (Figure 1). Consistent with our previous findings, these results suggest that activation of NFκB occurs in the amnion epithelial layer as a prelude to the onset of labour where it can drive the upregulation of pro-labour genes such as COX-2 (PGHS-2) and IL-8. This pre-labour activation of NFκB appears to be persistent since it is maintained in cells in primary culture for up to 48 hours and contrasts with uterine myocytes in primary culture in which no activity of NFκB is seen without stimulation (eg by IL-1β) irrespective of whether the cells were collected before or during labour [13]. Persistent or committed activation of NFκB in amnion is logical since the amnion, as part of the fetal membranes, is expelled following delivery with the placenta. In contrast, persistent activation of NFκB in myometrium would be detrimental since it would presumably lead to post delivery myometritis.

Activation of inducible NFκB pathways occurs typically through one of three pathways; canonical, non-canonical or atypical activation, yet the mechanism governing the NFκB activation pathways in the amnion prior to labour is not clearly elucidated. We examined protein levels of numerous key modulators of NFκB activation using immunoblotting of nuclear protein extracts of amnion samples. Consistent with canonical activation of NFκB levels of nuclear p65 in the pre-labour amnion cells were highly correlated to levels of nuclear phosphorylated p65 indicative of the phosphorylation-dependant translocation of p65 to the nucleus (Figure 1D). However, high correlation between nuclear p65 (both non-phosphorylated and phosphorylated) and nuclear Rel-B was also observed and this is consistent with non-canonical activation of NFκB (Figure 2A). However, if non-canonical activation was responsible for NFκB amnion activation we would expect to see highly correlated levels of nuclear p52 (dimerized to Rel-B during non-canonical activation) however no correlation between nuclear Rel-B and p52 was detected (Figure 2D). No positive correlation was observed between cytoplasmic IκBα with either nuclear p65 or phosphorylated p65 meaning that NFκB activation could not be attributed to atypical signalling pathways.

Although the most commonly characterised NFκB activation pathways do not seem to be responsible for pre-labour amnion activation, we consistently observed a striking correlation between nuclear p65 and pp65 with Rel-B (R^2^ = 0.8157 and R^2^ = 0.6288). Using immunoprecipation, we explored the possibility that these subunits may interact physically through protein/protein interactions in both basal and activated amnion cells. For the first time, we have shown that Rel-B and p65 interact in the nucleus of amnion epithelial cells (Figure 3A). This complex degrades quickly in the first 30 min of IL-1β stimulation before gradually increasing and peaking at 4 h. Similarly, binding of nuclear Rel-B to the NFκB consensus binding sequence using a non-radioactive DNA binding assay kit (TRANSAM) was maximal at 4 h. Rel-B contains a transcriptional activation domain but has the capacity to act as both a positive promoter of NFκB-dependent gene expression as well as a repressor of NFκB activity [26]. Few Rel-B target genes have been reported but its positive transcriptional role has been described through the use of the Rel-B^−/−^ mouse. These mice lack the thymic medulla and a class of dendrytic cells suggesting a critical role for Rel-B in the development of secondary lymphatic organs [27]. Interestingly, while wild type fibroblasts lack TNF-α activity, fibroblasts isolated from Rel-B^−/−^ mice do [28]. This suggests that one role of Rel-B is to epigenetically silence the TNF-α gene. Consistent with a role in mediating inflammation, Rel-B appears to play a role in mediating early innate immune responses to that of prolonged adaptive innate immune responses by down regulating acute inflammation and activating the maturation of dendritic cells necessary for antigen presentation and T-cell activation [29]. The transcriptional role of Rel-B/p65 complexes in pre-labour amnion epithelial cells remains to be elucidated, their presence in the nucleus and interaction with the NFκB consensus binding sequence along with their ability to modulate transcription warrants future investigation.

To garner a better understanding of the gene regulation underpinning amnion activation prior to labour onset, we then undertook a series of studies in which amnion epithelial cells were collected from a range of women undergoing elective caesarean section before the onset of labour. These cells were established in primary culture and their degree of NFκB activation was assessed directly and as a function of a downstream measure of NFκB activation; COX-2 mRNA and protein expression. COX-2 is an established NFκB dependent gene and marker of amnion activation. Prostaglandins produced via COX-2 facilitate cervical ripening and its increase in activated amnion samples is consistent with it being a critical player in the pathways leading to labour onset. Two distinct groups of samples were identified and classified into either low or high NFκB activity was (Figure 4). Interestingly, we did not observe a strong correlation between COX-2 mRNA and proteins levels in a number of samples. For example, high levels of COX-2 mRNA detected in sample “high 2” were not translated into high protein levels in this sample. In contrast, comparatively low levels of COX-2 mRNA were observed in sample “high 3” yet high levels of COX-2 protein were detected in this sample. This may reflect differences in post transcriptional stability between samples, however it is more likely that high mRNA and low COX-2 levels within samples represents increased processing of COX-2 toward prostaglandin production. cDNA microarray analysis was then performed to identify the cohort of genes upregulated in association with amnion activation. The resulting gene expression data was examined using PCA and hierarchical clustering to confirm that the underlying variance in the data was sufficient to classify the samples into two distinct groups (Figure 5). This step essentially validated the classification of amnion samples used on the basis of downstream NFκB-regulated genes.

A table of the top 20 genes significantly upregulated with amnion activation (Table 1) as well as a list of inflammatory genes upregulated with amnion activation (Table 2) were generated. We found a high increase in the expression of OTR (24-fold increase) in the fetal membranes upon activation. This is consistent with the concept that OTR is regulated through NFkB and is an important labour-associated gene in amnion [30] as in myometrium [31], [32]. The role of oxytocin and its receptor in the amnion is not as well established as in the myometrium since the amnion is non-contractile, however, oxytocin has been shown to stimulate prostaglandin production in rabbit amnion where OTR expression increases 200-fold at the end of pregnancy [33]. Moreover, myometrial contractions can be indirectly modulated by prostaglandin production by the deciduas [34], uterine endometrium and amnion cells [35]. This has therapeutic implications in that future tocolytics such as oxytocin receptor antagonists would likely require, in addition to myometrial contractility suppression, the ability to reach the amnion, chorion and decidua in order to inhibit oxytocin induced prostaglandin production and fetal membrane activation. Small molecule OTR antagonists might therefore have an advantage over OTR peptide analogues.

Ingenuity Pathway Analysis of the microarray data revealed two major networks associated with amnion activation. The first of these networks- cell death, cancer and morphology- is consistent with marked increase in apoptosis observed with the preparation of the amnion for rupture [36], [37] via the delamination and apoptosis of amnion epithelial cells (Figure 6). However, this observation contrasts with the well described role for NFκB as an anti-apoptotic transcription factor [38]. There exists known communication between the apoptotic pathway and NFκB through TNF receptor 1-associated protein TRADD [39] and in cancer tumours, NFκB induced upregulation of COX-2 drives the synthesis of prostaglandins that in turn stimulate prostaglandin receptors and activates cell proliferation and angiogenesis [40]. Consistent with a role in angiogenesis, we observed upregulation of CTGF (connective tissue growth factor, 14.9-fold increase) and CYR61 (cysteine-rich angiogenic inducer, 6.9-fold increase) in activated amnion samples. Similarly, the cell motility modulating genes THBS1 (thrombospondin, 2.7-fold increase), TUBB3 (tubulin beta 3, 3.2-fold increase) and TUBB2A (tubulin beta 2A, 4.5-fold increase) were found to be upregulated with amnion activation. Collectively these results suggest that amnion activation involves NFκB mediated apoptotic and angiogenic pathways which may act to maintain the integrity of the amnion during the hypoxic stress of labour and protect the fetus prior to delivery.

The second major gene network implemented in amnion activation was cell-to-cell signalling and interaction, DNA replication, recombination, repair and cellular development (17 focus genes- Figure 7). Much cross talk between cell-to-cell signalling and inflammatory response pathways exist and a number of additional genes involved in mediating inflammation were also observed in this study (Table 2). A 7.5-fold increase in transforming growth factor alpha (TGFα), which positively regulates epidermal growth factor (EGF) receptor activity [41], [42] was detected in activated amnion samples. Similarly, we identified a 4.6-fold increase in EGF epiregulin. EGF has previously been reported to upregulate COX-2 in amnion WISH cells [43]. Thus it reasonable to suggest the powerful positive feedback loops of pro-inflammatory cytokines of NFκB and vice versa, EGF on COX-2 and TGFα on EGF may account for the massive upregulation of COX-2 in the amnion activated samples.

Our results describe a complex network of signalling pathways involved in amnion activation, many of which are regulated by NFκB. We have presented a list of genes associated with amnion activation that will serve as an important database for future work and may provide a map for rational therapeutic design to prevent preterm labour and premature preterm rupture of the membranes.

## Materials and Methods

### Patient cohort and Ethics Statement

Approval for the study was obtained from the research and ethics committees of the Imperial College Healthcare NHS Trust and the Imperial College and all clinical investigation was conducted according to the principles expressed in the Declaration of Helsinki. Following informed written consent, intact fetal membranes were obtained from women undergoing elective Caesarean section at term prior to the onset of labour (n = 12) or following labour (n = 8). We took prelabour samples only from women whose indication for caesarean section was breech presentation, previous caesarean section or maternal request at between 39 weeks and term in whom there were no contractions. We took post-labour samples only from women who had established in spontaneous labour and had not been given oxytocin or prostaglandin to induce or augment labour. None of these patients had pre-eclampsia, gestational diabetes or any other complication of pregnancy.

### Amnion cell preparation

Amion cells were prepared from tissue as we have previously described [44]. Briefly, amnion was separated from the placenta and chorion, rinsed in PBS then cut into strips before incubating in 0.5 mM EDTA in PBS for 15 min. Strips were rinsed in PBS then digested with 2.5 mg/ml Dispase (Life Technologies, Paisley, UK) for 40 min at 37°C. Amnion epithelial cells, were dissociated by shaking vigorously in DMEM supplemented with 10% fetal calf serum (FCS, Sigma, Poole, UK). The cell suspension was centrifuged and the resulting pellet cultured in DMEM supplemented with 10% FCS, 1% L-glutamine and 1% penicillin-streptomycin at 5% CO_2_. For IL-1β treatments, cells were first serum starved for 16 h before 1 ng/ml IL-1β was added and incubated for 15, 30, 60 or 120 min.

Amnion samples were characterised as low, medium and high ‘activation’ based upon the level of nuclear localisation of NFκB p65 as assessed by western analysis. For microarray analysis of changes in gene expression samples were further refined into low and high activation groups based upon both nuclear localisation of NFκB p65 and expression of COX-2 measured by qRT-PCR and western analysis.

### Protein extraction and SDS-PAGE

Isolation of cytosolic proteins was achieved by lysing cells in 10 mM HEPES, 10 mM KCl, 0.1 mM EGTA, 0.1 mM EDTA, 2 mM 1–4 Dithrothreitol (DTT) and complete protease inhibitor (Roche, Hertfordshire, UK) diluted according to manufacturer's instructions. Lysates were centrifuged for 30 s at 12 000 *g* at 4°C and the supernatant collected. Nuclear proteins were isolated by resuspending the resulting pellets in buffer consisting of 10 mM HEPES, 10 mM KCl, 0.1 mM EGTA, 0.1 mM EDTA, 2 mM DTT, 400 mM NaCl and 1% (v/v) NP-40 and complete protease inhibitor. Samples were shaken vigorously for 15 min on ice then centrifuged 12 000 *g* at 4°C. In the case of whole cell protein extraction, confluent cell monolayers were scraped and lysed in RIPA buffer. Protein concentrations were determined using the Bio-Rad protein assay reagents (Bio-Rad Laboratories, Hertfordshire, UK) Approximately 40 µg of cytosollic and 30 µg of nuclear protein were resolved on pre-cast 10% Tris-Glycine gels (Invitrogen Life Technologies, Paisley, UK) and separated electrophoretically at 140 V (constant voltage) until the bromophenol blue dye migrated to the bottom of the gel.

### Western Blotting

Following SDS-PAGE separation proteins were transferred to Hybond C nitrocellulose membrane (GE Healthcare, Little Chalfont, UK) at 200 mA (constant current) before being blocked in 5% skim milk in phosphate-buffered saline (PBS) supplemented with 0.01% Tween 20 (PBS-T) for 1 hour. Membranes were then incubated with antibodies for p65 (1∶1000), phospho-p65 (1∶2000; Cell Signaling Technology, Danvers, MA, USA), Rel B (1∶500), p50 (1∶2000), p52 (1∶2000; Cell Signaling Technology) and IκBα (1∶2000) for 1 h at room temperature in blocking buffer containing 1% skim milk. Unless otherwise indicated, all antibodies were purchased from Santa Cruz Biotechnology (Santa Cruz, CA, USA). Membranes were then washed five times (5 min per wash) in PBS-T and then incubated for 1 h with the appropriate secondary antibody at room temperature. Immunoreactive protein was detected using enhanced chemiluminescence (GE Healthcare) and film exposure. Membranes were stripped (2% SDS, 62.5 mM Tris-HCl, pH 6.7 and 100 mM 2-mercaptoethanol) for 30 min at 50°C before being washed and reprobed for β-actin as a loading control.

### Coimmunoprecipitation

Non-labouring amnion epithelial cells were cultured to 90% confluence, serum starved overnight and stimulated with IL-1β for 30 min, 1 h, 4 h or 24 h. Cells were then extracted in a Nonidet P-40 (NP40) based buffer (150 mm NaCl, 50 mm Tris-HCl, 5 mm EDTA, 0.5% NP40). Co-immunoprecipitation was carried out by incubating the 300 µg whole cell extract with NFκB p65 conjugated A/G sepharose beads overnight. As a control, samples were also incubated in the presence of non-specific IgG preparations. Samples were then washed three times in lysis buffer before adding 30 µL of LDS loading buffer and boiling for 10 min. The samples were then loaded onto a 6% polyacrylamide gel, transferred to nitrocellulose as previously described and probed with antibodies raised against phospho-p65 and Rel-B.

### RNA extraction, Real-time PCR and microarray studies

Total RNA (1 µg) was isolated and used as a template for reverse transcription. Expression levels of each gene was determined by real-time PCR using an ABI PRISM 7700 sequence detection system according to manufacturer's instructions (PE Applied Biosystems, Forster City, CA). Specific primers were designed for Taqman with the primer express program (PE Applied Biosystems). Acquired data were analysed using Sequence Detector version 1.7 (PE Applied Biosystems) and were normalised to ribosomal L-19.

For microarray analysis RNA was extracted using the Qiagen RNeasy® Mini kit (Qiagen, Crawley, UK) according to the manufacturer's instructions and the concentration and purity determined by measurement of the OD260 and OD280 on a spectrometer. Samples were then sent to Almac Diagnostics (Craigavon, UK) for whole genome analysis using Affymetrix U133 arrays (High Wycombe, UK). For this, 100 ng of total RNA was used for cDNA in the first synthesis using the GeneChip® Expression 3′-Amplification Two-Cycle cDNA synthesis kit in conjunction with the GeneChip® Eukaryotic PolyA RNA Control kit (Affymetrix). Cleanup of the double-stranded cDNA was performed using the GeneChip® Sample Cleanup Module, which was followed by amplification and labelling using the GeneChip® Expression 3′-Amplification IVT Labelling Kit. Labelled cRNA was fragmented before 15 µg was hybridised for 16 h at 45°C. Finally, the array was washed and stained on a GeneChip® fluidics station 450 and subsequently scanned using a GeneChip® Scanner 3000.

### Statistical Analyses

Unless otherwise stated, results are presented as mean ± SEM. Results were log-transformed as the raw data was not normally distributed and were analysed by repeated measures analysis of variance with a *post-hoc* Bonferroni correction. *P*-values<0.05 were considered to be significant.

For microarray data, interpretation of results was carried out using log of ratio. Data was subject to a series of quality assurance filters including a gene expression level filter based on the cross gene error model, which leverages the observed variability of many expressed genes to estimate measurement precision (set to a minimum value of 15.47), a fold-change (minimum value of 1.5) and a confidence filter (*P*<0.05). The fold-change value of 1.5 was specifically selected to ensure minimise loss of potentially important and relevant biologically changes [45]. The expected proportion of incorrectly rejected null hypotheses (type 1 errors) borne out of multiple comparisons was corrected using the Bejamini and Hochberg False Discovery Rate. A total of 919 genes passed this filter and constituted the stringent gene list.

Principal Components Analysis (PCA) was utilised to examine inherent variation in the sample groups. In PCA, the first principal component represents of the largest amount of correlated variation in the data set. The second principal component (representing the second largest amount of correlated variation in the data set) is placed orthogonally to this and the resulting score plot was examined for any clustering trends. Hierarchical clustering using the average linkage method was used to further demonstrate relationships between gene expression levels in the samples. Software used for data QC was Genespring v 7.0 (Agilent Technologies Inc., CA, USA).

To identify canonical pathways and gene ontology groups for the gene expression data, Ingenuity Pathway Analysis (IPA) softward (Ingenuity Systems, Redwood City, CA) was utilised.
